# GDP prediction of The Gambia using generative adversarial networks

**DOI:** 10.3389/frai.2025.1546398

**Published:** 2025-03-05

**Authors:** Haruna Jallow, Alieu Gibba, Ronald Waweru Mwangi, Herbert Imboga

**Affiliations:** ^1^Department of Mathematics (Data Science Option), Pan African University Institute for Basic Sciences, Technology and Innovation, Kiambu, Kenya; ^2^Center for Policy, Research and Strategic Studies (CepRass), Kanifing, Gambia; ^3^Department of Computing and Information Technology, Jomo Kenyatta University of Agriculture and Technology, Kiambu, Kenya; ^4^Department of Statistics and Actuarial Sciences, Jomo Kenyatta University of Agriculture and Technology, Kiambu, Kenya

**Keywords:** algorithms, GANs, GDP growth, machine learning, prediction, The Gambia

## Abstract

Predicting Gross Domestic Product (GDP) is one of the most crucial tasks in analyzing a nation’s economy and growth. The primary goal of this study is to forecast GDP using factors such as government spending, inflation, official development aid, remittance inflows, and Foreign Direct Investment (FDI). Additionally, the paper aims to provide an alternative perspective to Generative Adversarial Networks method and demonstrate how such deep learning technique can enhance the accuracy of GDP predictions with small data and economy like The Gambia. We proposed the implementation of Generative Adversarial Networks to predict GDP using various economic factors over the period from 1970 to 2022. Performance metrics, including the coefficient of determination R^2^, mean absolute error (MAE), mean absolute percentage error (MAPE), and root- mean-square error (RMSE) were collected to evaluate the system’s accuracy. Among the models tested—Random Forest Regression (RF), XGBoost (XGB), and Support Vector Regression (SVR)—the Generative Adversarial Networks (GAN) model demonstrated superior performance, achieving the highest accuracy, which is 99% prediction accuracies. The most dependable model for capturing intricate correlations between GDP and its affecting components, however, RF and XGBoost, also achieved an accuracy of 98% each. This makes GAN the most desirable model for GDP prediction for our study. Through data analysis, this project aims to provide actionable insights to support strategies that sustain economic boom. This approach enables the generation of accurate GDP forecasts, offering a valuable tool for policymakers and stakeholders.

## 1 Introduction

Macroeconomic indicators are crucial in various aspects of modern society. They serve as essential tools in economic governance, enabling politicians to assess the overall health of the economy. Similarly, these indicators have become instruments for citizens to evaluate the performance of their elected officials. Accurate interpretation of numerical data and statistical analysis is fundamental in today’s world, with macroeconomic indicators often at the heart of such assessments. Prominent indicators such as inflation, public deficit, GDP growth, and unemployment are central for policymakers to make informed decisions (Evholt and Larsson, 2020). Macroeconomic indicators and economic forecasts are critical in the contemporary financial system. These indicators focus on forecasting economic trends, which help individuals, institutions, and governments make decisions about liabilities, employment, spending, trade, investment, and crucial policies that drive economic activities. The ability to accurately predict these economic indicators serves as a guiding tool for decision-making and promoting growth.

When analyzing macroeconomic indicators, there is no universally accepted method for constructing models or performing calculations. Approaches vary across countries and evolve over time. Additionally, there is ongoing debate regarding whether some methods disproportionately benefit certain groups or unfairly evaluate governance practices (Evholt and Larsson, 2020). This study does not take a position on the most appropriate way to define or calculate macroeconomic indicators, nor does it suggest that GDP is more or less important than other indicators. Such discussions are left to Economists. Instead, this research is focused on developing a high-quality dataset and an effective model.

GDP is an essential indicator for assessing a nation’s economic performance and overall well-being. It provides a comprehensive evaluation of all products and services generated in a given economy, reflecting the total economic activity of the country, including investment, government spending, and private consumption. By tracking GDP fluctuations, policymakers and other stakeholders can determine whether an economy is expanding or declining. Accurate GDP forecasts are critical for governments to develop economic strategies and set achievable goals. A reliable GDP forecasting model supports proactive decision-making for sustainable growth by offering valuable insights into future economic conditions.

Time series forecasting, particularly GDP prediction, has been a focal point of research for decades, bridging the interests of industry and academia. Many real-world processes inherently follow a time-series structure, especially in finance and economics, where indicators like GDP growth, inflation rates, remittance inflows, and other macroeconomic metrics are pivotal. Accurate GDP forecasting is critical for informed decision-making in financial planning and policy development. Traditionally, GDP forecasting has relied on econometric models, which use historical data to predict future trends. While effective in simpler economic environments, these methods often fall short in today’s increasingly complex and interconnected global economy. They tend to rely on point estimates and fail to account for uncertainty in predictions. In contrast, machine learning offers a powerful alternative, capable of processing vast datasets and uncovering intricate patterns that traditional linear models often miss.

A country’s economic development is impacted by several elements, such as its social, economic, and cultural environment. For small, developing nations like The Gambia, understanding the dynamics of GDP growth is vital for achieving sustainable progress. Various elements, such as remittance inflows, foreign direct investment, official development assistance, trade metrics (exports and imports), inflation, and government spending, shape The Gambia’s economy. Alongside demographic factors like population growth and human capital, these elements create a complex network that impacts GDP. Consequently, devising a comprehensive approach that integrates multiple factors is crucial for developing an effective GDP forecasting model.

This study utilizes annual data from 1970 to 2022, sourced from the World Bank Database, to explore advanced forecasting methods. A combination of deep learning techniques, such as Generative Adversarial Networks (GANs), and classical machine learning models, including Random Forest (RF), Support Vector Machines (SVM), and XGBoost, is employed to uncover patterns and relationships that traditional econometric approaches may overlook. This aligns with emerging research demonstrating the superiority of machine learning for managing non-linear patterns and high-dimensional datasets (Rizzato et al., 2023; Yu, 2022).

Generative Adversarial Networks (GANs) which were first proposed by Goodfellow et al. (2014), have emerged as a cutting-edge approach to forecasting, particularly in economics and finance, where traditional models often struggle with non-linear relationships and adapting to rapidly changing conditions. This is especially true for small economies like The Gambia, where limited datasets and complex economic dynamics pose unique challenges. GANs address these gaps by learning intricate patterns and generating realistic data distributions, making them ideal for capturing the effects of diverse economic factors such as government spending, inflation, remittance inflows, and foreign direct investment. Moreover, GANs can be retrained with updated data, ensuring adaptability and continuous improvement, which is crucial for timely and accurate forecasting in dynamic economic contexts.

Evholt and Larsson (2020) demonstrated the potential of GANs for macroeconomic forecasting by predicting U.S. unemployment rates, outperforming traditional ARIMA models across all forecasting horizons. Their study also explored the integration of unconventional data sources, such as Twitter, combined with Natural Language Processing (NLP) techniques like DistilBERT. While these attempts did not surpass benchmarks in unemployment forecasting, they showed the value of social media data in improving predictions for the more erratic and trend-sensitive S&P 500 index. This highlights the potential of hybrid approaches combining GANs with NLP for macroeconomic predictions.

Vuletic et al. (2024) further advanced the application of GANs by introducing an innovative, economics- driven loss function tailored for financial time series forecasting. This novel loss function adapts GANs for supervised learning tasks, enabling them to provide probabilistic forecasts that go beyond traditional point estimates. Their results, based on equity data, demonstrated that GANs with this loss function outperform classical models like LSTMs and ARIMA, especially in terms of generating more accurate and uncertainty-aware predictions. Their approach achieved higher Sharpe Ratios, illustrating the value of GANs not only in prediction accuracy but also in risk management and decision-making.

Building on these advancements, our study applies GANs to the complex task of GDP prediction for The Gambia, a small economy with limited historical data and intricate economic dynamics. The GAN-Econ architecture introduced in this work is inspired by the studies of Evholt and Larsson (2020) and Vuletic et al. (2024). By incorporating an economics-driven loss function, our model enhances regression accuracy and addresses the challenges posed by limited data. Similar to the approaches of Evholt and Larsson (2020), we explored how GANs can capture non-linear patterns and generate robust predictions in data-constrained environments. Additionally, like Vuletic et al. (2024), we leveraged GANs’ ability to provide probabilistic forecasts, addressing uncertainty—an essential aspect of economic forecasting. This research contributes to the expanding body of literature on GAN-based forecasting by applying these advanced methodologies to GDP prediction in The Gambia, marking one of the first attempts to utilize adversarial training in this context. By demonstrating the effectiveness of GANs in capturing complex economic relationships, this study provides valuable insights for policymakers and stakeholders aiming to enhance economic resilience and informed decision-making.

This study aims to transcend the limitations of classical methods by leveraging the potential to improve GDP prediction accuracy. A particular focus is placed on addressing the challenges of forecasting in a small open economy like that of The Gambia, characterized by limited economic data. Classical machine learning and time-series methods often assume rigid priors about the target variable’s distribution, constraining their ability to capture uncertainty—a critical aspect for applications in risk management, policymaking, and financial planning. By integrating advanced deep learning techniques, this work also seeks to bridge the gap between economists and policymakers. By addressing deficiencies in traditional economic modeling and data analysis, the study aims to support The Gambia’s journey toward economic resilience and sustainable growth. Additionally, it contributes to the growing body of literature comparing the predictive accuracy of conventional time-series models and modern deep learning approaches like GANs, offering insights into their relative strengths and applicability in economic forecasting. This research not only advances the understanding of GDP prediction but also underscores the importance of adopting innovative tools to navigate the complexities of modern economies.

To achieve this goal, we developed a novel deep learning-based architecture named Generative Adversarial Networks for Economics (GAN-Econ), specifically designed for GDP prediction in The Gambia. GAN- Econ incorporates an innovative, economics-driven loss function within the generator to improve regression performance. By leveraging modern data analysis techniques and robust machine learning methodologies, this study aims to enhance the accuracy of economic forecasts, thereby supporting the development of more effective economic policies and financial decision-making frameworks Thilaka and Sundaravalli (2024) and Janamolla and Syed (2024). Our approach benchmarks GAN-Econ trained using binary cross-entropy (BCE) loss, against traditional machine learning models such as Random Forest (RF), Support Vector Machines (SVM), and XGBoost (XGB). The primary objective is to evaluate whether GANs can outperform classical machine learning models in delivering accurate and reliable GDP forecasts. Experimental results indicate that GANs significantly improve prediction accuracy, particularly in scenarios with limited datasets, as they effectively address challenges posed by small and sparse data. This adaptability makes GAN- Econ particularly suited for applications in economies like The Gambia, where data availability is often constrained. To the best of our knowledge, this research represents the first application of adversarial training in GDP analysis and forecasting, marking a significant advancement in the field of economic modelling and prediction.

Summary of Main Contributions:

- Introduction of GAN-Econ, a novel GAN-based architecture for GDP prediction in The Gambia, with an economics-driven loss function to improve regression performance.
- GAN-Econ addresses challenges of data scarcity and non-linear relationships in small, complex economies through adversarial training, providing reliable GDP forecasts.
- Benchmarking GAN-Econ against classical machine learning models (Random Forest, SVM, XGBoost) demonstrates superior performance, particularly in data-limited scenarios.
- The study highlights the potential of GANs for overcoming limitations of traditional econometric models, offering a flexible and adaptive approach for continuous improvement in economic forecasting.

This paper’s remainder is organized as follows: Section 2 reviews previous studies on GDP forecasting methods. The methodology is illustrated in Section 3. Section 4 outlines the theory/calculation. Section 5 covers the result analysis. Section 5 presents the discussion of results. Lastly, Section 5 offers the conclusion and recommendations for the research.

## 2 Related work

This part provides an overview of related studies concerning methods for GDP prediction and the generative adversarial network.

### 2.1 Methods for predicting gross domestic product

Based on research conducted in this domain, the techniques used to address GDP prediction challenges can generally be categorized into two main approaches.

The first approach includes econometric models, encompassing traditional econometric techniques for forecasting GDP. The second approach consists of models based on soft computing. Soft computing refers to methods inspired by biological processes, including artificial intelligence techniques such as artificial neural networks (ANNs).

Common econometric techniques include the autoregressive model (AR), moving average model (MA), autoregressive moving average model (ARMA), and autoregressive integrated moving average model (ARIMA) (Zhou et al., 2018) are examples of common econometric approaches. In general, each new signal is treated by these models as a noisy linear mixture of separate noise components and previous signals. But, many of these models operate under strict assumptions, such as the independence and identical distribution (i.i.d.) of noise terms or specific distributional properties (e.g., t-distribution). Real-world financial data, however, often fail to adhere strictly to such assumptions. To address these limitations, (Pellegrini et al., 2011) presented the GARCH model, which is a generalized autoregressive conditional heteroscedasticity model for conditional variances. They predicted financial time series using the ARIMA-GARCH model.

Conventional methods for macroeconomic forecasting frequently overlook energy-related aspects embedded in economic datasets, which may result in inaccuracies in predicting GDP. The research by Zhang et al. (2023), conducted recently, addresses this limitation by examining the interplay between energy big data and economic indicators. It utilizes a deep learning-based long short-term memory (LSTM) neural network augmented with wavelet analysis (WA) techniques. This methodology dissects macroeconomic variables and incorporates energy-economic coupling features to develop a predictive framework. The integrated LSTM-WA model outperforms standard models, particularly in handling intricate, nonlinear, and multi-variable datasets, delivering improved precision and enhanced generalization for quarterly GDP forecasts.

Machine learning techniques have revolutionized economic forecasting by enabling precise predictions of GDP growth, inflation trends, and stock market dynamics using historical data. The research by employs advanced regression analysis and machine learning methods, including linear regression, decision trees, and neural networks, in combination with feature engineering and data preprocessing strategies like data normalization and addressing missing values. The results indicate that machine learning models surpass traditional econometric methods, especially for short-term forecasting. Neural networks demonstrate superior performance in forecasting GDP growth and inflation trends, while decision tree models prove more effective for predicting stock market movements. The study emphasizes the significance of data quality, periodic model updates for long-term reliability, and advocates for integrating real-time data and combining deep learning with econometric techniques in future research. It highlights the practical applications of machine learning in shaping economic policies and analyzing financial markets, enhancing the accuracy of economic forecasts.

In order to anticipate the actual U.S. GDP, the study of Maccarrone et al. (2021) assesses the forecasting skills of several models. They find that the machine learning K-Nearest Neighbor (KNN) model outperforms conventional time series techniques and successfully captures the self-predictive nature of the U.S. GDP by using quarterly data from 1976 to 2020. To improve forecasting accuracy, they looked into predictors like the yield curve, its latent components, and a variety of macroeconomic factors. Only when longer forecast horizons are taken into account do the predictions show improvement. Using machine learning algorithms provides more information for making data-driven decisions.

To forecast Japan’s real GDP growth between 2001 and 2018, the study of Yoon (2021) creates machine learning models, namely a random forest model and a gradient boosting model. The predictions are contrasted with standards from the Bank of Japan and the International Monetary Fund. The mean absolute percentage error and root mean squared error metrics are used to assess forecast accuracy, while cross- validation is used to optimize hyperparameters. The results show that both machine learning models perform better than the benchmark predictions, with the gradient boosting model outperforming the random forest model in terms of accuracy. The study emphasizes how machine learning methods might be incorporated into macroeconomic forecasting.

GDP is a vital economic indicator, essential for analyzing a country’s economy and growth. Gharte et al. (2022) study aims to offer an alternative to traditional econometric methods by demonstrating how machine learning techniques can enhance the accuracy of GDP predictions. Various social, economic, and cultural factors influencing GDP were analyzed from 1970 to 2018, and supervised learning methods were employed to develop predictive models. Three algorithms were used to assess the models’ performance, with Gradient Boosting achieving the highest accuracy, followed by Random Forest and Linear Regression. The final model was integrated into a web application capable of estimating a nation’s GDP using characteristics supplied by users.

Hossain et al. (2021) the primary goal is to predict GDP growth using parameters such as GDP per capita, inflation rate, government debt, total investment, remittances, and unemployment rate. Machine learning algorithms are utilized to uncover complex relationships between these factors and GDP growth, enabling more accurate predictions. This project aims to connect the general public and economists with valuable economic insights, offering a means to identify strategies for achieving desired GDP growth. It also helps highlight which factors most significantly influence GDP growth, which have minimal impact, and which contribute to its decline. By providing analyzed data, the system supports setting economic goals, understanding socio-economic scenarios, and implementing informed actions to sustain higher GDP growth rates.

Chu and Qureshi (2023) study assesses many forecasting techniques, such as deep learning (DL) and machine learning (ML), to forecast the growth of the US GDP. By employing a recursive forecasting strategy, it assessed performance across different timeframes using three datasets: nine important variables out of a larger group of 224 predictors, and an extended subset that included high-frequency business data. The analysis explored how the choice of predictors and modeling techniques influenced forecast accuracy. The findings highlight that density-based machine learning techniques, like boosting and bagging, slightly outperformed sparsity-based techniques like Lasso for short-term forecasts, particularly when there are many predictors. However, their advantage diminished for longer-term forecasts. Simpler models leveraging strong high-frequency predictors were more effective for long-term predictions than complex ML and DL models using low-frequency data. Ensemble ML methods consistently outperformed DL models, showcasing their strength in economic forecasting tasks. GDP is a critical indicator of a nation’s economic health and quality of life, reflecting the value of products and services generated over a specific time period. Thilaka and Sundaravalli (2024) study uses machine learning to predict GDP, employing predictors like Life Expectancy, Human Development Index (HDI), CO2 emissions, and service worker percentages. The model, developed in Python using the Gapminder dataset, analyzes these factors to provide insights into economic performance. Three machine learning algorithms—Decision Tree Regression (DTR), Support Vector Regression (SVR), and Polynomial Regression (PR)—were tested, focusing on non-linear regression due to the dataset’s structure. The Decision Tree Regression model emerged as the most accurate, achieving an R-squared value of 1. The study shows how machine learning may be used to predict economic indicators like GDP with accuracy.

GDP is a crucial indicator of a country’s financial stability, reflecting its economic condition per capita. The COVID-19 pandemic has caused sudden fluctuations in GDP, necessitating further analysis to understand and predict these changes. Sa’adah and Wibowo (2020) study explores deep learning methods as a solution for GDP prediction, focusing on Long Short-Term Memory (LSTM) and Recurrent Neural Networks (RNN). The research findings show that both LSTM and RNN models effectively fit the actual GDP data, achieving prediction accuracies between 80 and 90%. These findings demonstrate the promise of deep learning methods techniques to monitor and understand GDP fluctuations, even during crises like the COVID-19 pandemic, offering valuable insights for economic stability and planning.

Using data from New Zealand, the study of Richardson and Mulder (2018) assesses machine learning algorithms’ real-time nowcasting capabilities. Using a wide range of current quarterly macroeconomic variables, several well-known machine learning algorithms are trained to nowcast real GDP growth for each quarter from 2009Q1 to 2018Q1. The predictive accuracy of these nowcasts is compared with that of traditional univariate and multivariate statistical models. The findings reveal that machine learning algorithms surpass traditional statistical models in predictive accuracy. Additionally, combining individual machine learning nowcasts enhances performance beyond that of individual nowcasts alone.

The article of Ahammad et al. (2024) delves into the importance of GDP and its growth, emphasizing the need for precise forecasting. It examines the role of machine learning algorithms in enhancing prediction accuracy. The article reviews multiple studies that demonstrate the effectiveness of machine learning methods, such as linear regression, random forest regression, linear regression, and autoregressive integrated moving average (ARIMA) in forecasting GDP. These algorithms process data, detect patterns, and generate accurate predictions, thereby aiding in better decision-making for economic analysis and planning.

Koch et al. (2024) study investigates how to calculate the GDP per capita of nations and regions in North America and Europe during the previous seven centuries using biographical information about historical personalities. The researchers created an elastic net regression model for feature selection by examining information such as the birthplaces, sites of death, and occupations of hundreds of thousands of historical figures. 90% of the variation in past income levels can be explained by this model, which also makes it possible to estimate GDP per capita for areas and time periods for which data is unavailable. Four proxies of economic output—urbanization rates, body height, well-being, and church building activity—were used to validate the model’s estimates. A historical reversal of fortune between Southwest and Northwest Europe between 1,300 and 1800, mostly due to Atlantic commerce, is clearly shown by the data. These results, which are provided in a comprehensive dataset with confidence intervals, show how biographical data can be used to augment historical GDP estimations.

Real-time GDP monitoring is essential for policymaking. Muchisha et al. (2021) studies used 18 quarterly macroeconomic and financial market factors to create and compare machine learning models that predict Indonesia’s GDP growth in real time. The ability of six well-known machine learning algorithms—Random Forest, LASSO, Ridge, Elastic Net, Neural Networks, and Support Vector Machines—to predict GDP growth between 2013:Q3 and 2019:Q4 was assessed. The Pearson correlation coefficient, RMSE, and MAD were used to gauge forecast accuracy. Every model fared better than the AR(1) benchmark, with Random Forest exhibiting the best performance on its own. Forecast combinations employing equal weighting and lasso regression were used to increase accuracy; Random Forest, Ridge, Support Vector Machine, and Neural Network models combined with lasso regression produced the best results.

Traditional GDP per capita calculations often relied on simplistic models, mainly using population figures, which led to imprecise predictions and limited the ability to make informed policy decisions. To address this, Naaz et al. (2024) proposed using machine learning (ML) to simulate the intricate, non- linear connections between different socio-economic and demographic factors, such as population, area, literacy, and climate to improve the accuracy of GDP per capita forecasting. Our study compares two ML models—Random Forest and Neural Network regression—using performance metrics like Mean Squared Error, Mean Absolute Error, and R-squared. This approach provides more accurate predictions, offering valuable insights for policymakers, economists, and researchers to make better decisions in economic planning and resource allocation.

GDP is a key indicator of an economy’s size and performance, with real GDP growth often used to assess overall economic health. Dwarakanath and Shivakumara (2022) project aims to predict global GDP and calculate its yearly growth using machine learning, while analyzing the fluctuating nature of GDP across years. By visualizing these fluctuations and identifying the factors affecting GDP, the project helps to understand the drivers of economic change. Additionally, it evaluates the significance of different features in GDP calculation, offering insights into the best and worst performing predictions for various countries. Kant et al. (2024) study evaluates the effectiveness of various econometric and machine learning techniques in nowcasting GDP in near real-time. Using Dutch GDP data from 1992Q1 to 2018Q4 and a comprehensive set of monthly indicators, the analysis examines forecast accuracy and the utilization of extensive macroeconomic and financial predictors. The findings revealed that, on average, the random forest method delivers the most precise forecasts and nowcasts, while the dynamic factor model excels in back casting accuracy.

GDP is a crucial measure for evaluating economic performance, guiding policy decisions, investment choices, and assessing social well-being. Predicting GDP is vital for shaping economic policies, business strategies, and financial markets. In the study of Ahammad et al. (2024), using 43 years of historical data from Wikipedia and the World Bank, machine learning techniques are utilized to predict Bangladesh’s GDP over the coming years. The dataset includes nine key features that significantly influence GDP. Various machine learning models, including Linear Regression, Random Forest, AdaBoost, KNN, Decision Tree, and Lasso, were tested, with KNN achieving the highest accuracy of 98.40%, followed by Random Forest, Decision Tree, AdaBoost, Linear Regression, and Lasso with accuracies of 95.80, 95.32, 95.05, 90.78, and 67.14%, respectively.

GDP is a key indicator of an economy’s size and performance, with real GDP growth commonly used to assess overall economic health. Velidi (2022) study aims to predict global GDP and its annual growth rate using machine learning techniques. Given the annual fluctuations in GDP, understanding the factors influencing it is essential. By visualizing these factors, the study simplifies comprehension. The research also evaluates the importance of these variables in GDP calculation, allowing comparisons of GDP growth rates across countries, highlighting the best and worst predictions. Various machine learning algorithms are employed to analyze global GDP in this study.

Machine learning algorithms have become increasingly popular for economic forecasting, and Srinivasan et al. (2023) study focuses on predicting India’s GDP using advanced methods. Linear and polynomial regression techniques were used to evaluate data from sources such as time series analysis and inflation rates. According to the results, polynomial regression worked more accurately than linear regression, with the polynomial model obtaining a prediction accuracy of 91% as opposed to 87% for the linear model. This illustrated how non-linear interactions between independent and dependent variables may be captured by the polynomial model. The study emphasizes how crucial high-quality data and sophisticated machine learning methods are to increasing the accuracy of economic forecasting. By using polynomial regression, the study provides valuable insights for companies and governments, as precise GDP forecasts. This research highlights the effectiveness of polynomial regression for predicting Indian GDP and sets a foundation for future studies in economic forecasting using machine learning.

### 2.2 Generative adversarial network

A generative model *G* that approximates the data distribution and a discriminative model *D* that assesses the probability that a sample comes from the training data rather than *G* are trained simultaneously in a Generative Adversarial Network (GAN), a system for learning generative models through an adversarial process. The goal of training G is to increase the likelihood that *D* will make a mistake.

Macroeconomic forecasting, typically addressed through time series analysis, has seen few efforts using machine learning, especially with unconventional data like social media. Evholt and Larsson (2020) thesis utilizes a Generative Adversarial Network (GAN) to predict U.S. unemployment, outperforming the ARIMA benchmark across various time horizons. Attempts to incorporate Twitter data with the Natural Language Processing (NLP) model DistilBERT show promising but not benchmark-beating results. Additionally, the models’ ability to forecast the US S&P 500 stock index is examined, where Twitter data enhances accuracy by reflecting current public discourse trends. These findings highlight the potential of social media data in macroeconomic predictions, particularly for volatile indicators like unemployment and stock market trends, suggesting future exploration of NLP-GAN models.

Srivastava et al. (2021) study explores using Generative Adversarial Networks (GANs) to forecast financial market variables. The framework employs Gated Recurrent Units (GRUs) as the generator and a Convolutional Neural Network (CNN) as the discriminator. Stock data for six companies from the Nifty 50 index—Hindalco, IOC, NTPC, ONGC, PowerGrid, and Wipro—were retrieved using the Yahoo Finance API to implement the model.

In the financial industry, stock price prediction is essential for developing successful stock exchange strategies. In order to forecast high-frequency stock market movements, the Zhou et al. (2018) study presents a flexible architecture that uses convolutional neural networks (CNN) and long short-term memory (LSTM) for adversarial training. The model makes use of publicly accessible indices from trade software, bypassing the need for complex financial theories and technical analyses, making it accessible to non- specialist traders. The study mimics actual trading scenarios and employs a rolling partition method for training and testing sets to assess how model update cycles affect forecast accuracy. Numerous tests showed that this method considerably lowers forecast mistakes and improves the accuracy of stock price direction predictions.

The amount of data that must be digitally saved and communicated has significantly increased as a result of the development of artificial intelligence. Strong cybersecurity defenses must be put in place since digital landscapes have intricate infrastructures. One kind of deep learning model that has shown promise in tackling changing security issues is Generative Adversarial Networks (GANs). The survey of Arifin et al. (2024) poll investigates how GANs might improve cybersecurity, namely in areas like malware identification, BotNet detection, mobile and network trespassing, and intrusion detection systems (IDS). Additionally, it talks about the difficulties in applying GANs in these situations and makes recommendations for future research, highlighting how GANs could improve cybersecurity.

Modelling in finance is a complex task due to the intricate statistical properties of the data and the unknowns behind its behavior. While deep learning algorithms are advancing, the potential uses of data- driven modelling are limited by the inadequacy of data to train these models. A neural network architecture called Generative Adversarial Networks (GANs) has demonstrated success in producing images and is currently being used to produce financial data, including time series. Rizzato et al. (2023) study provides an overview of GANs, exploring their capacities, limitations, and practical applications in finance. Through testing three popular GAN architectures on financial time series, the study found promising results in terms of statistical properties. Overall, GANs have advanced significantly in the banking industry and can be a useful tool for data scientists working in this area.

The study of Vuletic et al. (2024) explores the application of Generative Adversarial Networks (GANs) for probabilistic forecasting of finance time series forecasting using a new, economically motivated loss function for the generator. This loss function enhances the suitability of GANs for classification tasks, positioning them within a supervised learning framework while generating complete conditional price return probability distributions based on past outcomes. Unlike traditional point estimates, our approach provides uncertainty estimates. Experiments on equity data demonstrate that our methodology outperforms classical models like LSTMs and ARIMA, achieving higher Sharpe Ratios.

Zhao et al. (2024) paper presents self-attention generative adversarial networks (SAGANs) are presented in this paper for the detection of credit card fraud, addressing challenges such as data imbalance, overfitting, and the difficulty of simulating fraudulent behavior. By utilizing self-attention mechanisms, SAGANs identify key patterns in transaction data, improving the understanding and detection of fraud. Incorporating generative adversarial networks (GANs) enables the generation of realistic fraudulent data, enhancing algorithms for detecting fraud. Empirical findings from several datasets of credit card transactions demonstrate that SAGANs outperform traditional methods in accuracy and recall, effectively capturing the complexity of fraud and improving detection performance.

While the application of Generative Adversarial Networks (GANs) has gained significant attention in various domains such as image generation, natural language processing, and even some financial applications, their use in economic forecasting, particularly for GDP prediction in small economies, remains largely unexplored. Most existing literature on GDP forecasting relies on classical time-series models or machine learning techniques like Random Forest (RF), Support Vector Machines (SVM), and XGBoost. These models, though effective, often assume a strong prior distribution for the target variable, limiting their ability to fully capture the uncertainty and complex interactions between macroeconomic indicators, especially in data-scarce contexts.

Furthermore, much of the existing research on GDP prediction tends to focus on developed economies with abundant data, neglecting the unique challenges faced by small economies like The Gambia. These studies often overlook the intricate relationships between socio-economic, demographic, and environmental factors that influence GDP, which are crucial for understanding the economic dynamics of developing nations. The use of a narrow set of predictors—such as government spending, inflation, and foreign direct investment—limits the predictive accuracy, as it fails to account for other vital factors such as remittance inflows, population dynamics, sectoral contributions (e.g., agroforestry, fishing), and indicators of human capital and urbanization.

Moreover, the majority of GDP prediction models rely on point estimates, which do not provide a measure of uncertainty, a critical component for effective decision-making in financial and policy settings. This gap in capturing uncertainty further limits the applicability of traditional methods in risk management and long-term planning. This study seeks to fill these gaps by introducing a novel deep learning-based approach—Generative Adversarial Networks for Economics (GAN-Econ)—for GDP forecasting in The Gambia. By incorporating a comprehensive set of socio-economic variables and utilizing adversarial training, this research addresses the limitations of traditional models in terms of both predictive accuracy and uncertainty estimation. GAN-Econ is tailored to small datasets, making it particularly suitable for economies with limited data availability. In doing so, this study not only advances the application of GANs in economic forecasting but also provides a more holistic and robust framework for predicting GDP in developing economies. This work represents a significant contribution to the growing body of research on probabilistic forecasting and deep learning applications in economic and financial contexts, offering valuable insights for policymakers and stakeholders in small economies.

## 3 Materials and methods

### 3.1 Research context

This study focuses on predicting the GDP growth for The Gambia using advanced machine learning techniques. The analysis covers the period from 1970 to 2022, a time frame chosen for its comprehensive coverage of economic trends and the availability of reliable data. This period allows for the capture of long-term patterns and variations in GDP growth, reflecting the country’s economic evolution over five decades. The Gambia, a small West African nation, faces unique economic challenges, including limited data availability, dependence on foreign aid, and a reliance on agriculture and remittances. These factors contribute to complex economic interactions, making GDP forecasting particularly challenging. The study’s emphasis on The Gambia provides valuable insights into economic planning and policy formulation for developing economies with similar characteristics.

### 3.2 Research design

This research adopts a quantitative methodology, leveraging machine learning techniques to predict GDP growth. Generative Adversarial Networks (GANs) were employed as the primary model due to their ability to generate realistic synthetic data and capture complex nonlinear relationships. Three additional models—Random Forest (RF), Support Vector Machine (SVM), and XGBoost—were used for comparative analysis. It utilizes 14 predictor variables, including government expenditure, foreign direct investment, remittance inflows, official development aid, exports, imports, population, and urbanization metrics. These variables were selected based on their established relevance to GDP dynamics, capturing critical economic and socio-economic determinants in The Gambia.

### 3.3 Data collection

The dataset was compiled from credible sources such as the World Bank Open Databases and Central Bank of The Gambia. These institutions provide reliable, annually recorded economic indicators essential for robust analysis. The selected time frame of 1970 to 2022 is representative of The Gambia’s economic trends, encompassing periods of growth, stagnation, and recovery. This range ensures a holistic understanding of the factors influencing GDP growth. The dataset comprises annual time-series data (1970–2022). It includes 14 independent variables and one dependent variable (GDP), ensuring sufficient depth for model training and evaluation.

### 3.4 Data preprocessing

Missing values were addressed using mean imputation, ensuring data completeness while preserving statistical properties. This method was chosen for its simplicity and effectiveness in handling small datasets. To standardize the dataset and ensure compatibility with the GAN architecture and the classical machine learning models, the Min-Max Normalization technique was applied. This approach scaled all features to a predetermined range, facilitating model convergence and improving performance. We scaled our data using the Min-Max Normalization approach as can been in Equation 1 below:


(1) $$x^{\prime }=\frac{x-\min\left(x\right)}{\max\left(x\right)-\min\left(x\right)}$$

Relevant features were identified using Pearson Correlation analysis. Variables with correlation coefficients ≥ 0.5 or ≤ −0.5 with GDP growth were selected. These included government expenditure, population, exports, imports, and secondary education enrolment, among others, ensuring that only the most impactful predictors were retained.

**Table:** 

Foreign_direct_investment	0.612886
ODAid_received	0.612776
Exports	0.814244
Imports	0.824819
Net_migration	0.626877
Population	0.835354
Agroforestry_fishingValue	0.882439
Government_expenditure	0.921720
Secondary_education	0.729167
Urban_population	0.812646
GDP_per_capita	1.000000

Following the completion of the above procedures, we divided our cleaned dataset into train-test segments in order to train our models using the our manin algorithm, Generative Adversarial Networks, and other three algorithms (Random Forest, XGBoosting, and Support Vectors Regressor). The dataset was divided into two subsets: 80% was allocated for training, 20% for testing: y train is used to capture the dependent feature” GDP,” while X train is used to collect the independent features.

### 3.5 Proposed methodology

**Figure 1:**
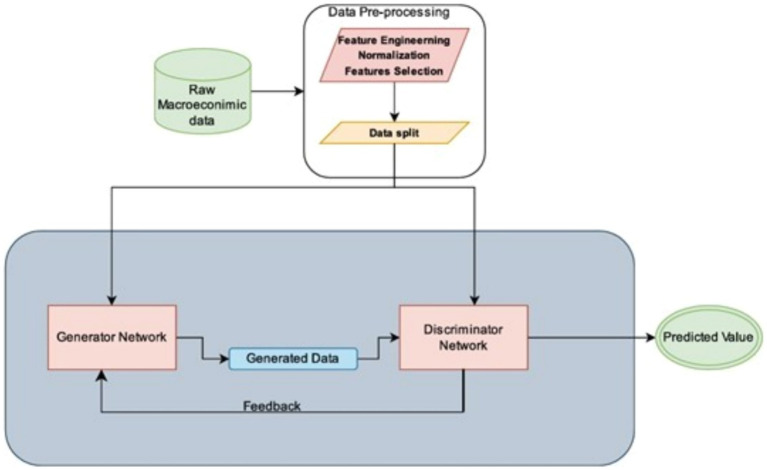
GAN implementation architecture.

This section outlines the problem formulation and presents the architecture of our proposed GAN framework for GDP prediction. The GAN consists of a generator, which synthesizes plausible GDP data, and a discriminator, which evaluates the authenticity of the generated data. Training involves an adversarial process, where the generator aims to deceive the discriminator while the discriminator learns to identify real versus synthetic data. Figure 1 illustrates the project’s GAN implementation architecture.

## 4 Theory/calculation

We give a quick overview of the machine learning techniques we used this study for GDP prediction.

### 4.1 Generative adversarial networks (GAN) overview

Ian Goodfellow and his associates first presented a set of generative machine learning frameworks known as generative adversarial networks, or GANs, in 2014 (Goodfellow et al., 2014). Generative Adversarial Networks (GANs) are a type of deep learning model designed to generate new data by learning from existing datasets. They attracted a lot of attention because of their efficacy and simplicity. The framework introduced in 2014 has advanced significantly in a short period of time, with significant advancements achieved in the original application of GANs—image generation—as well as the creation of hundreds of new types of GANs to optimize for a variety of tasks, from computer vision to bank fraud detection.

Although these frameworks are still relatively new in comparison to other domains, there are several applications to finance and financial time series analysis, which makes for an interesting field of study. They are particularly effective in cases where data is scarce or highly nonlinear, making them well-suited for economic applications such as GDP prediction. In machine learning (ML), GANs are a member of the generative model family. More formally, a generative model is a statistical model of the joint probability distribution on $P\left(X|Y\right)$ , this refers to a method capable of generating new data points based on an observed variable $\left(X\right)$ and a corresponding target variable $\left(Y\right)$ . In most cases, the procedure entails identifying and learning patterns in the input data, enabling the model to generate new samples while maintaining the characteristics of the original dataset. The two components of the GANs Adapted for GDP Prediction are:

The Discriminator functions as a classifier trained on historical economic data, such as GDP values, government spending, and inflation rates. Its primary role is to differentiate between real economic data (from the training dataset) and synthetic data generated by the Generator. The Discriminator evaluates the authenticity of the data and outputs a binary result: 1 if the data is real and 0 if it is synthetic. This mechanism enables the Discriminator to learn patterns and relationships inherent in the actual economic data, improving its ability to detect discrepancies in the synthetic outputs. On the other hand, the Generator is designed to create new, realistic economic data points. Starting with a random noise vector as input, it generates synthetic GDP-related data that reflects plausible economic trends and values. The ultimate goal of the Generator is to produce data so realistic that the Discriminator cannot reliably distinguish it from real data. Through iterative feedback from the Discriminator, the Generator continuously refines its outputs, capturing complex patterns and relationships within the economic indicators to enhance the accuracy of GDP predictions.

**Figure 2:**
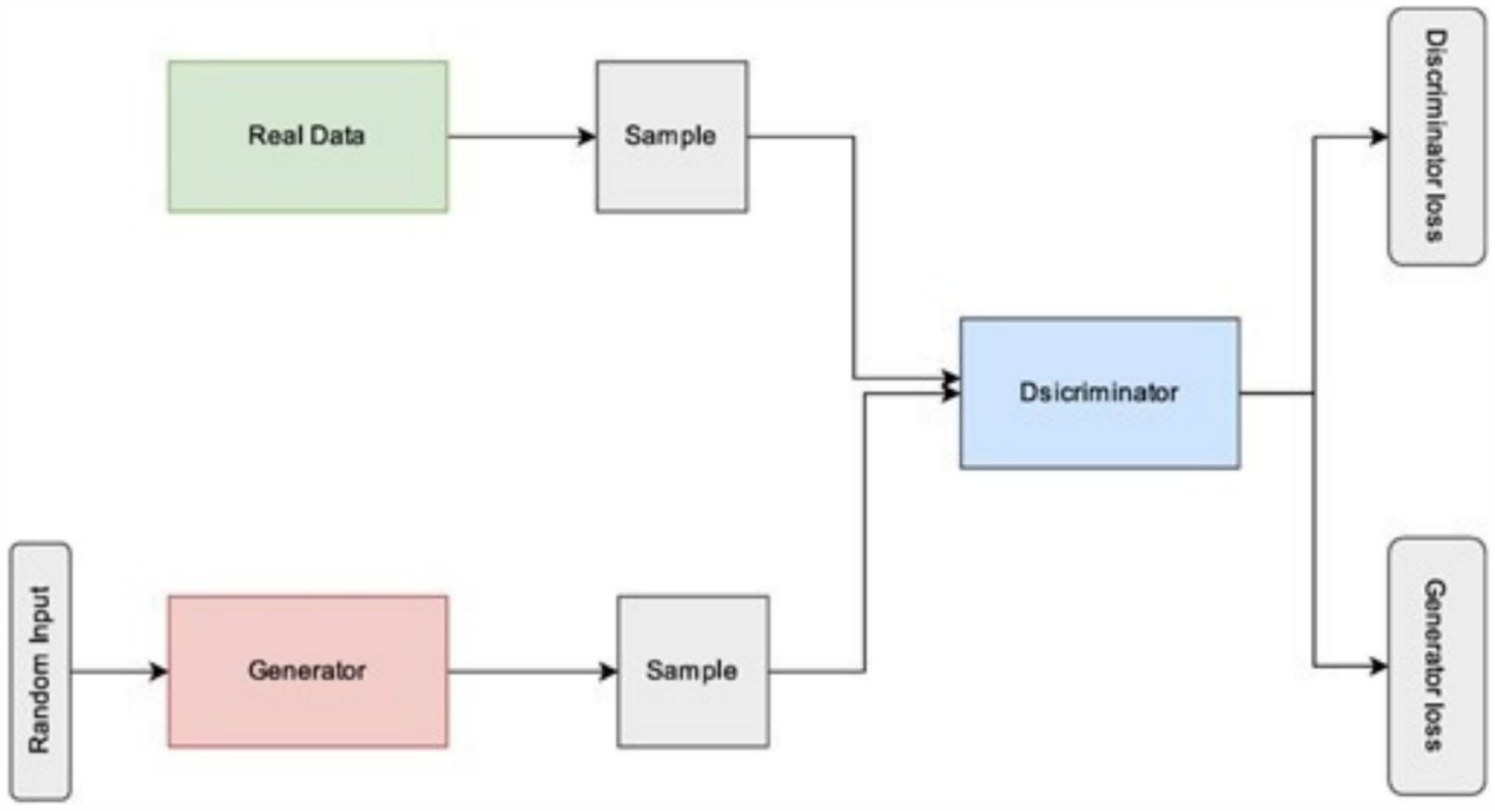
Generative adversarial networks architecture.

As seen in Figure 2 and explained below, two models, usually neural networks, are trained simultaneously in the original GAN framework, which estimates generative models using an adversarial process: A generative model called the Generator (G) records the distribution of data and creates new instances. In the second model, a classifier called the Discriminator (D) determines the probability that a sample came from the training data instead of G. In order to increase the possibility that the Discriminator will incorrectly classify its output, the Generator is taught.

*Cost/Loss function:* Minimax objective function (Value Function or Cost Function of Minimax Game played by Generator and Discriminator) as shown in Equation 2:


(2) $$\begin{array}{c} \min\\ G \end{array}\;\begin{array}{c} \max\\ D \end{array}V\left(D,G\right)=E_{\left(x\right)}\left[\log D\left(x\right)\right]+E_{\left(z\right)}[\log\left(1-D\left(G\left(z\right)\right)\right)$$

where:

- The discriminator’s estimate of the likelihood that genuine data instance x is real is denoted by $D_{x}$ .
-
$E_{x}$ is the expected value over all instances of actual data.
- When fed noise *z*, the generator’s output is denoted as $G\left(z\right)$ .
- The discriminator’s estimate of the likelihood that a phony instance is real is $D\left(G\left(z\right)\right)$ .
-
$E_{z}$ is the expected value over all random inputs to the generator, or more specifically, the expected value across all fictitious instances that are generated $G\left(z\right)$ .

While the Generator is in charge of producing data, the Discriminator is responsible for assessing the quality of the generated data and providing input. These neural networks are optimized under game-theoretic conditions: The discriminator’s job is to determine the source of the input, which could be the generator or the actual data set. The generator’s job is to create data that will fool the discriminator.

The noise is generated using a standard normal distribution $N\left(0,1\right)$ via the torch.randn function. This ensures that the noise has a mean of 0 and a standard deviation of 1, providing a well-defined statistical structure to aid in stable training. The noise vector (*z*) is generated for each data sample in the batch, with its size determined by the batch size and the latent dimension (latent dim = 10. The noise is then concatenated with the input features (*x*) before being passed to the generator, enabling the model to leverage both the randomness of the noise and the structured information from the input features. This combination helps the generator create diverse and contextually relevant outputs, as the noise introduces variability while the input features provide meaningful context.

While the noise generation process itself is not unique, its integration with the input features is a distinguishing aspect of your model. By combining noise with input features, the generator can model both the inherent variability and the structured relationships in the economic data. Additionally, using GANs with noise for a regression task, rather than traditional tasks like image generation, is relatively unique. This approach helps the model capture uncertainty in real-world data, making it particularly suited for predicting economic indicators where randomness and variability play a significant role.

GANs are highly suited for GDP prediction, especially for economies like The Gambia, where historical GDP data may be limited. By generating realistic synthetic data, GANs can augment the dataset, enhancing model training and predictive accuracy. They excel in modelling complex, nonlinear relationships among economic indicators such as inflation, remittances, and government spending, capturing intricate dependencies that traditional models may overlook. Furthermore, GANs are adaptable to small economies, effectively generating realistic outputs tailored to unique economic patterns, making them an ideal tool for predicting GDP in contexts with distinct dynamics like The Gambia.

### 4.2 Support vector machine regression (SVM) overview

The SVM algorithm looks for a function *f* (*x*) that, for all training data, has a maximum divergence from the targets, *y_i_*. If the function is linear, it is defined as shown in Equation 3.


(3) $$f\left(x\right)=\left(w,x\right)+b$$

where *b* is the bias, *x* is the input vector, and w is the weight vector. Finding this linear function while making sure it is as” smooth” as feasible is the goal of the SVM algorithm. However, it’s possible that there is not a function that meets these requirements. Regression mistakes can therefore occur up to the values of $\xi_{i}$ and ${\xi_{i}}^{*}$ because the procedure permits slack variables, $\xi_{i}$ . Furthermore, the cost function incorporates an extra term, w , that minimizes the coefficients on the explanatory variables in order to penalize non-smoothness. Next, Equations 4, 5 provides the support vector regression’s objective:


(4) $$\min0.5{\left\|w\right\|}^{2}+C\overset{l}{\underset{i=1}{\sum }}\left(\xi_{i}+{\xi_{i}}^{*}\right)$$

(5) $$\mathrm{subject}\;to\;\{\begin{array}{c} y_{i}-\left(w_{i},x_{i}\right)-b\leq \xi +,{\xi_{i}}^{*}\\ \left(w,x_{i}\right)+b-y_{i}\leq \xi +,{\xi_{i}}^{*}\; \end{array}$$

The regularization parameter, or constant term *C*, regulates the trade-off between penalizing overfitting and minimizing mistakes. With a radial basis function (RBF) kernel function, we employed linear epsilon insensitive SVM (-SVM) regression for our modeling. As is customary in the literature, we employed an epsilon value that is one tenth of the target variable’s interquartile range as a stand-in for the training sample’s noise level. Similarly, the target variable’s interquartile range is used to set the hyperparameter *C*.

### 4.3 Random Forest Regressor overview

An ensemble learning technique called Random Forest (RF) mixes several decision trees, each of which is constructed using a randomly selected subset of data. Each tree is generated independently, following the same distribution, ensuring diversity within the forest Breiman (2001). Unlike the bagging technique, RF introduces randomness during tree construction by choosing a random subset of predictors at each split, RF adds unpredictability to the tree-building process, ensuring no interaction between trees during the training process. Because of its versatility, RF can be used for problems involving both regression and classification. When used in regression, RF averages the predictions of each individual tree to forecast the result.

Regression trees are the basic building component of RF. One response variable and *p* predictors for *N* observations are included in each of the many binary regression trees that RF combines, each of which is built using bootstrap samples of the dataset. Consider a training dataset *L* consisting of $\left(x_{i},y_{i}\right)$ for i=1,2,…N , where $\left(x_{i}=x_{i1},x_{i2},\ldots x_{ip}\right)$ . Determining the splitting variables, splitting points, and general structure of the tree are all necessary while building a regression tree. The following are the steps involved in creating the trees Hastie et al. (2005):

1. Suppose we partition the dataset *L* into *M* regions denoted by $R_{1},R_{2},\ldots R_{M}$ .
2. The response within each region $R_{m}$ is modeled as a constant value $c_{m}$ :

$$f\left(x\right)=\overset{M}{\underset{m=1}{\sum }}c_{m}I\left(x\in R_{m}\right)$$

3. The predicted value $c_{m}$ is obtained by averaging $y_{i}$ for all observations within region $R_{m}$ :

$${\hat{c}}_{m}=ave\left(y_{i}|x_{i}\in R_{m}\right)$$

To identify the best binary partition, the algorithm evaluates various threshold values for potential splits and selects the threshold that minimizes the sum of squared errors. As shown in Equation 6, for example, in regions *R*_1_ and *R*_2_, the algorithm identifies the splitting variable *j* and splitting point *s* by solving:


(6) $$\begin{array}{c} \min\\ j,s \end{array}\left[\begin{array}{c} \min\\ c_{1} \end{array}\underset{x_{i}\in R_{2}\left(j,s\right)}{\sum }{\left(y_{i}-c_{2}\right)}^{2}+\begin{array}{c} \min\\ c_{2} \end{array}\underset{x_{i}\in R_{2}\left(j,s\right)}{\sum }{\left(y_{i}-c_{2}\right)}^{2}\right]$$

The dataset is split into two subsets that correspond to the split once the best split has been determined. Each node becomes a terminal node when this process is completed iteratively, and each node meets the user-defined minimum node size.

### 4.4 XGBoost algorithm overview

A strong and scalable gradient boosting system intended for effectiveness and performance is called XGBoost (eXtreme Gradient Boosting). Similar to conventional gradient boosting, XGBoost sequentially minimizes a given loss function to create an ensemble of weak learners, usually regression trees. The algorithm enhances predictive performance by adding new trees to correct the errors of previous iterations, guided by a loss function. XGBoost introduces several advanced regularization techniques, including $L_{1}$ (Lasso) and $L_{2}$ (Ridge) penalties, to prevent overfitting and improve model generalization. Additionally, it incorporates efficient handling of sparse data and missing values, along with optimized system-level enhancements like parallelization, tree pruning, and distributed computing. XGBoost is based on the principle of gradient boosting, where new models are sequentially trained to fix the earlier models’ residual faults. The approach optimizes an objective function that incorporates a regularization term to avoid overfitting as well as the loss function, which gauges how well the model fits data. The goal is provided by:


(7) $$Obj=\overset{n}{\underset{i=1}{\sum }}L\left(y_{i},{\hat{y}}_{i}\right)+\overset{k}{\underset{j=1}{\sum }}\Omega \left(f_{j}\right)$$

where L is the loss function, Ω is the regularization term, $y_{I}$ is the actual value, and ${\hat{y}}_{i}$ is the predicted value.

In the objective function presented in Equation 7, the variable in the first component, $L\left(y_{i},{\hat{y}}_{i}\right)$ , is ${\hat{y}}_{i}$ , which represents the predicted value generated by the model. This component evaluates the difference between the actual value $y_{i}$ and the predicted value ${\hat{y}}_{i}$ using a specified loss function (e.g., mean squared error for regression or log-loss for classification). The goal is to minimize this loss during training to improve the model’s predictive accuracy.

In the second component, $\Omega f_{j}$ , the variable is $f_{j}$ , which denotes the j−th tree in the ensemble. This term quantifies the complexity of the model by penalizing the structure of each tree, such as its depth or the number of leaf nodes. By incorporating $\Omega f_{j}$ into the objective function, the algorithm enforces regularization, which helps prevent overfitting and ensures better generalization to unseen data.

### 4.5 Model evaluation

Evaluating the performance of a GAN during training can be challenging, as the ultimate goal is to generate realistic samples that closely resemble the real data distribution. For GDP growth prediction, the evaluation process for our models involved both qualitative and quantitative measures to ensure the stability and accuracy of the generated results.

Qualitative evaluation involved visually inspecting and comparing the generated GDP samples to the real data from the training set. This allowed us to assess how closely the synthetic data aligned with historical GDP trends. By analyzing the visual patterns, we ensured that the GAN-generated data exhibited realistic characteristics, such as smoothness and logical economic progression. Quantitative evaluation utilized several metrics to assess the quality and reliability of the generated GDP predictions. Following our models’ training, the stability and prediction accuracy of GDP growth were evaluated using yearly data spanning from 1970 to 2022. Four analysis metrics, as shown in Equations 8–11 were employed to assess the quality of predictions:

Root Mean Square Error (RMSE):


(8) $$\mathrm{RMSE}=\sqrt{\frac{1}{n}\overset{n}{\underset{i=1}{\sum }}{\left(y_{i}-{\hat{y}}_{i}\right)}^{2}}$$

RMSE provides a measure of the average magnitude of errors in GDP predictions, with a focus on larger deviations.

Mean Absolute Error (MAE):


(9) $$MAE=\frac{1}{n}\overset{n}{\underset{i=1}{\sum }}|y_{i}-{\hat{y}}_{i}|$$

MAE quantifies the average absolute difference between actual and predicted GDP values, offering a straightforward interpretation of prediction errors.

Mean Absolute Percentage Error (MAPE):


(10) $$\mathrm{MAPE}=\frac{100\%}{n}\overset{n}{\underset{i=1}{\sum }}|\frac{y_{i}-{\hat{y}}_{i}}{{\hat{y}}_{i}}|$$

MAPE evaluates the prediction accuracy in percentage terms, highlighting relative errors.

Coefficient of Determination ( $R^{2}$ ):


(11) $$R^{2}=1-\frac{\sum_{i=1}^{n}{\left(y_{i}-{\hat{y}}_{i}\right)}^{2}}{\sum_{i=1}^{n}{\left(y_{i}-{\bar{y}}_{i}\right)}^{2}}$$

$R^{2}$ indicates the proportion of variance in GDP data explained by the model, providing an overall measure of fit.

## 5 Result

In this section, we examined our models’ predictive power and determine which model best fits our dataset. The experimental setup and tools used to implement the models are described, and evaluate the models are discussed. It also explains the process of hyperparameter tweaking and its outcomes. We also assessed comparative analysis of the models’ results, conducted a scenario analysis and finally, we displayed the study’s ultimate findings.

### 5.1 Experimental setup

We used a computer with an Intel Cor(TM)i7-7600U CPU running at 2.80 GHz and an integrated Intel^®^ HD Graphics 620 GPU for the testing phase. With 16 GB of RAM, the system had more than enough processing capability to train and assess the models. Python 3.12.0 was used for the experiments, and TensorFlow v2.16.1 was used to create deep learning models. Other libraries were used, including Scikit- learn for preprocessing and assessment, Pandas for data manipulation, and Matplotlib for visualization. The Jupyter Notebook environment was used for the experiments.

### 5.2 Tuning Hyperparameters

The procedure of hyperparameter tweaking is essential. The best set of parameter values to increase a model’s accuracy cannot be determined with certainty. Four distinct models have been used in this investigation; To perform better, each requires optimization of a vast number of parameters. To determine the optimal parameter combinations, we advise employing a grid search technique along with a trial-and-error approach. Tables 1, 2 shown the hyperparameters used for each our of models.

**Table 1:** Model names and hyperparameter tuning settings.

Model name	Hyperparameters for model
Random forest regressor	‘n estimators’: [100, 300, 500] ‘max features’: [‘auto’, ‘sqrt’, ‘log2’] ‘max depth’: [None, 10, 20, 30] ‘min samples split’: [2, 5, 10] ‘min samples leaf’: [1, 2, 4] ‘bootstrap’: [True, False]
XGBoost	‘learning rate’: [1, 0.1, 0.01, 0.001] ‘n estimators’: [100, 500, 1,000] ‘max depth’: [3, 5, 8] ‘subsample’: [0.7, 1] ‘colsample bytree’: [0.7, 1] ‘min child weight’: [1, 5, 10] ‘gamma’: [0, 0.1, 0.2]
Support vector regressor	‘C′: [1, 10, 100, 1,000] ‘gamma’: [1, 0.1, 0.01, 0.001, 0.0001] ‘kernel’: [‘rbf’] ‘epsilon’: [0.1, 0.01, 0.001] ‘shrinking’: [True, False]
GAN	‘latent dim’: [5, 10, 20] ‘epochs’: [200, 500, 1,000] ‘batch size’: [16, 32, 64] ‘learning rate’: [0.0002, 0.0001, 0.00005] ‘beta 1’: [0.5, 0.9] ‘beta 2’: [0.999, 0.9999] ‘activation function’: [‘LeakyReLU’, ‘ReLU’, ‘Sigmoid’]

**Table 2:** Optimal hyperparameters.

Model name	Optimal hyperparameters
Random forest regressor	‘n estimators’: [100] ‘max features’: [‘sqrt’, ‘log2’] ‘max depth’: [20] ‘min samples split’: [2] ‘min samples leaf’: [1] ‘bootstrap’: [False]
XGBoost	’learning rate’: [0.1] ‘n estimators’: [500] ‘max depth’: [3] ‘subsample’: [1] ‘colsample bytree’: [1] ‘min child weight’: [1] ‘gamma’: [0]
Support Vector Regressor	’C′: [1000] ‘gamma’: [0.1, 1] ‘kernel’: [‘rbf’] ‘epsilon’: [0.1] ‘shrinking’: [True]
GAN	‘latent dim’: [10] ‘epochs’: [1000] ‘batch size’: [16] ‘learning rate’: [0.0002] ‘beta 1’: [0.5, 0.9] ‘beta 2’: [0.999, 0.9999] ‘activation function’: [‘LeakyReLU’, ‘ReLU’, ‘Sigmoid’]

### 5.3 Forecasting results

The dataset was split in four criteria: *all features without scaling, all features with scaling, selected features without scaling, and lastly selected features with scaling* and three machine learning algorithms (RF, SVR and XGB) were trained and evaluated on this four criteria. The main deep learning algorithm (GAN) was trained and evaluated on only two of the four criteria (*all features with scaling and selected features with scaling*). Tables 3, 4 provides an overview of the models’ forecasting performance throughout the 1970–2022 sample period.

**Table 3:** Evaluation results of different models without optimization.

Algorithm	Features	Scaling	MAE	RMSE	MAPE	R^2^ Score
SVR	All	No	219.34	268.44	1.248	−0.169
	All	Yes	222.42	272.13	1.265	−0.201
	Selected	No	219.07	268.10	1.247	−0.166
	Selected	Yes	221.16	270.84	1.259	−0.190
Random forest	All	No	30.61	33.40	0.116	0.982
	All	Yes	40.75	46.53	0.181	0.965
	Selected	No	28.66	32.02	0.105	0.983
	Selected	Yes	38.39	44.62	0.174	0.968
XGBoost	All	No	27.81	37.14	0.068	0.978
	All	Yes	35.81	43.88	0.095	0.969
	Selected	No	26.62	31.84	0.073	0.984
	Selected	Yes	31.93	42.98	0.099	0.978
GAN	All	Yes	0.341	0.402	0.231	0.952
	Selected	Yes	0.067	0.074	0.078	0.982

**Table 4:** Evaluations of Models Performance with Optimization.

Algorithm	Features	Scaling	MAE	RMSE	MAPE	R^2^ Score
SVM	All	No	235.44	251.33	1.1099	−0.0246
	All	Yes	51.44	60.70	0.2192	0.9402
	Selected	No	235.44	251.33	1.1099	−0.0246
	Selected	Yes	55.73	60.54	0.2201	0.9405
Random forest	All	No	23.56	29.83	0.0826	0.9856
	All	Yes	43.13	51.43	0.1584	0.9571
	Selected	No	20.13	28.87	0.0677	0.9865
	Selected	Yes	38.15	45.87	0.1480	0.9659
XGBoost	All	No	25.49	31.02	0.0859	0.9844
	All	Yes	33.87	41.13	0.1017	0.9726
	Selected	No	25.50	31.13	0.0669	0.9843
	Selected	Yes	30.61	41.79	0.0926	0.9717
GAN	All	Yes	0.1147	0.1429	0.1154	0.9828
	Selected	Yes	0.0499	0.0617	0.0543	0.9968

The GAN model emerged as the most effective predictive tool among the evaluated models. When trained with selected features and scaling, it achieved an exceptional R^2^ score of 0.9968, indicating that it explained nearly all the variance in GDP growth. Additionally, it recorded the lowest MAE (0.0499) and RMSE (0.0543), suggesting that its predictions closely matched the actual GDP growth values. The GAN model also demonstrated the smallest MAPE (0.0617%), reflecting minimal percentage error. The GAN’s ability to outperform traditional models can be attributed to its architecture, which excels in capturing complex, nonlinear relationships within the data. The iterative interplay between the generator and discriminator enabled the creation of high-quality synthetic data, augmenting the training dataset and improving model generalization. These advantages were particularly beneficial in addressing the challenges posed by The Gambia’s small and sparse dataset.

XGBoost also delivered strong performance, particularly when trained with selected features without scaling. It achieved an R^2^ score of 0.9843, along with a low MAE (0.0669) and RMSE (0.031.13). The model effectively captured the upward and downward trends in GDP growth, as evidenced by its near-alignment with actual values in the time-series plots. However, XGBoost fell slightly short of the GAN model in predictive accuracy, highlighting the added value of the GAN’s synthetic data generation capabilities. The Random Forest model performed well, achieving an R^2^ score of 0.9865 with selected features and no scaling. It demonstrated low MAE (0.0677) and RMSE (0.028.87), showcasing its robustness in capturing the variability of GDP growth. Despite its strong results, the model’s reliance on ensemble averaging may have limited its ability to capture subtle nonlinear patterns compared to the GAN model.

The SVM model struggled to predict GDP growth accurately, with a negative R^2^ score (−0.0246) in most configurations, indicating a poor alignment between actual and predicted values. Even after optimization, its best performance yielded an R^2^ score of only 0.9405, with higher MAE and RMSE values compared to the other models. The SVM’s challenges can be attributed to its sensitivity to data sparsity and outliers, which may have hindered its effectiveness in this study.

### 5.4 Comparative analysis

**Figure 3:**
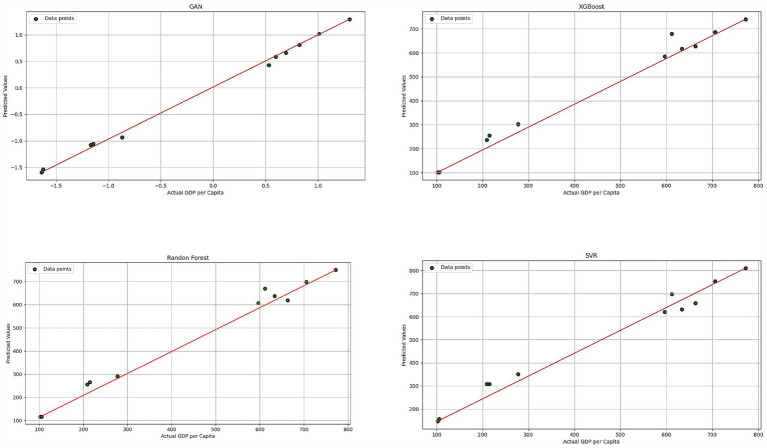
Scatter plots of actual GDP per capita vs. predicted values for each ML method.

The evaluation results (The results shown in Tables 3, 4) revealed that the GAN model consistently outperformed the other three models across all metrics, particularly when trained with selected features and scaling. The scatter plots (Figure 3) showed an almost perfect 45-degree alignment for the GAN model, with minimal scatter among data points, underscoring its superior predictive accuracy. In contrast, the other models exhibited some deviation from the actual values, with the SVM model showing the most significant mismatches.

**Figure 4:**
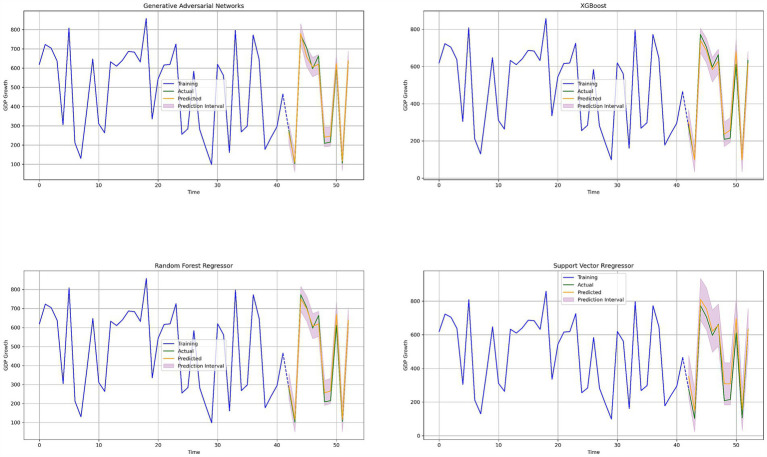
Annual GDP growth nowcasts in real time for every ML technique.

Time-series plots (Figure 4) further highlighted the GAN model’s ability to closely track actual GDP growth trends, with its predictions nearly overlapping the actual data throughout the study period. While XGBoost and Random Forest also captured the general trends, they displayed slightly larger deviations in certain periods, particularly during periods of rapid economic change.

### 5.5 Scenario analysis using the random forest

While predictive accuracy is a cornerstone of economic modelling, interpretability plays an equally critical role, particularly when the objective is to derive actionable insights for policymakers. For this reason, the Random Forest (RF) model was chosen over the Generative Adversarial Networks (GANs) model for scenario analysis and result interpretation.

The RF model demonstrated superior interpretability by providing clear and intuitive insights into the relative importance of various economic factors influencing GDP growth. Its feature importance analysis allowed for the identification of key drivers, such as government expenditure, trade dynamics, and urbanization, making it particularly well-suited for evaluating the impact of hypothetical economic scenarios. In contrast, while GANs excel in predictive accuracy, their complex architecture, involving the interplay between a generator and a discriminator, makes them less interpretable. This complexity poses challenges in isolating the specific factors contributing to predictions, limiting their utility for scenario analysis, where understanding the causal relationships between variables is essential.

By leveraging the RF model, the scenario analysis provided actionable insights into how changes in economic factors affect GDP growth. For example, the model highlighted the benefits of favourable trade conditions in a high-exports, low-imports scenario and the mixed impacts of urbanization paired with reduced government expenditure. Such clarity and the ability to pinpoint influential variables are vital for crafting targeted economic policies. Thus, the RF model was not only effective in capturing the relationships between economic variables but also in translating these relationships into interpretable and practical insights, justifying its selection for scenario analysis and interpretation over GANs.

#### 5.5.1 Scenario 1: high exports, low imports

In this scenario, exports were increased by 50%, while imports were reduced by 50%. This represents an economic environment with enhanced export activity and reduced reliance on imports, potentially stimulating domestic production and GDP growth. The Random Forest model predicted a slight increase in GDP growth under these favorable export–import conditions. However, the results indicated that while exports significantly contribute to growth, other factors, such as inflation or investment levels, could moderate these benefits. The results for scenario one is MAE = 25.70 RMSE = 34.77 R^2^ = 0.976.

#### 5.5.2 Scenario 2: high urban population, low government expenditure

This scenario simulated a 40% increase in the urban population alongside a 40% reduction in government expenditure. It reflects a setting where urbanization accelerates, but government spending on public services and infrastructure declines. The Random Forest model predicted mixed impacts on GDP growth. While urbanization could drive economic activity, the reduction in government expenditure might constrain growth by limiting public investment. The results for scenario one is MAE = 67.94, RMSE = 99.27, R^2^ = 0.807.

#### 5.5.3 Scenario 3: baseline (current) conditions

The baseline scenario maintained current levels of exports, imports, government expenditure, and urban population. This scenario provided a reference point to evaluate the impact of the changes in Scenarios 1 and 2. The model forecasted moderate GDP growth, reflecting the relative stability of the current economic conditions. The results for scenario one is MAE = 18.19, RMSE = 27.50, R^2^ = 0.985.

**Figure 5:**
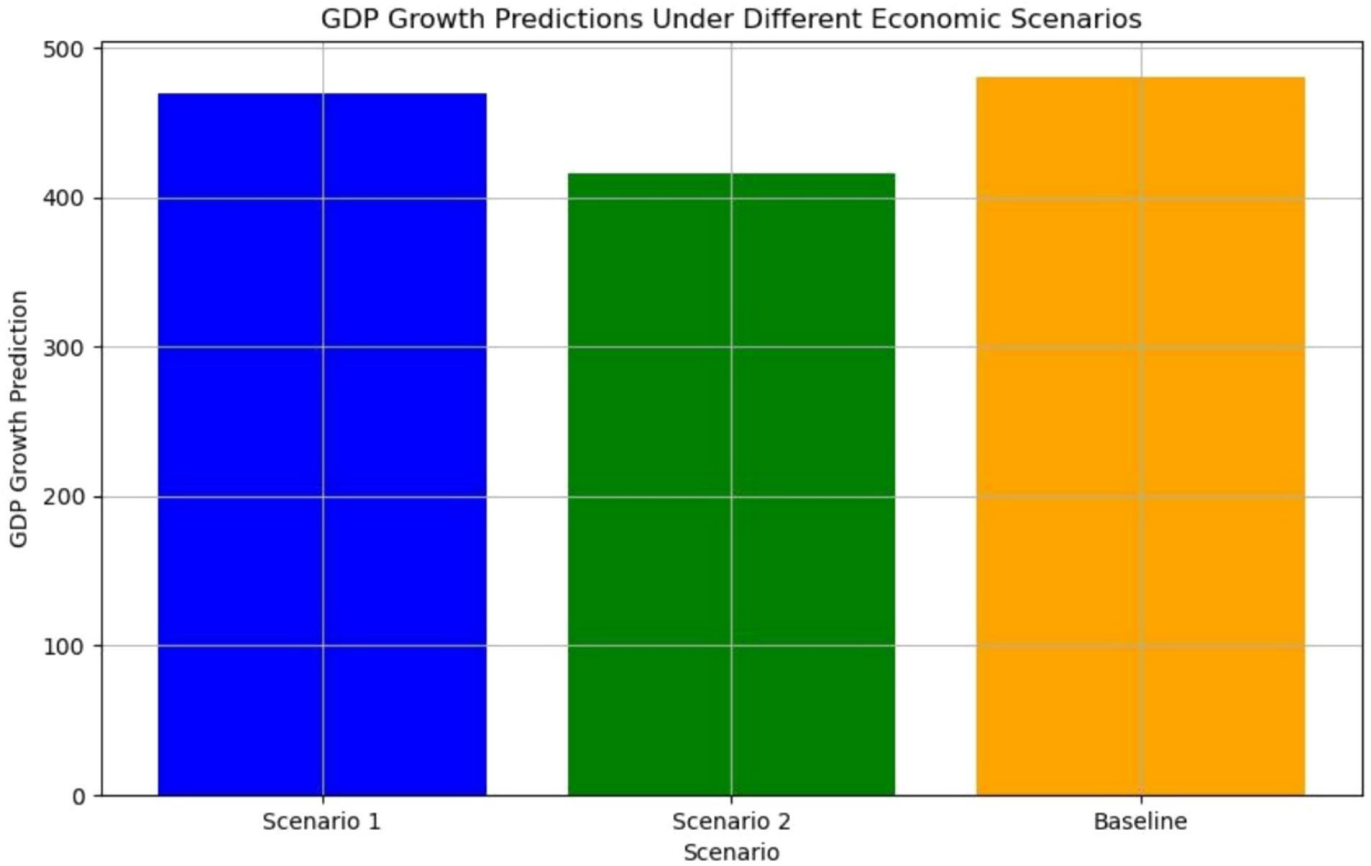
GDP predictions under varying scenarios.

Figure 5 illustrates the forecasted GDP under these varying scenarios, highlighting the shifts in anticipated economic performance. The baseline scenario yielded the highest predicted GDP growth, reflecting the stability of current economic conditions. Conversely, Scenario 2, characterized by high urban population growth and reduced government expenditure, resulted in the lowest GDP predictions. This underscores the importance of maintaining balanced public investment to sustain economic growth, even amidst urbanization. These findings emphasize the utility of scenario analysis in identifying the economic trade- offs associated with policy decisions and provide evidence-based insights for crafting strategies to optimize GDP growth.

### 5.6 Results comparisons

We reviewed several studies but unfortunately, only a few were directly relevant to our work. However, we found two studies, “GDP Growth Prediction of Bangladesh using Machine Learning Algorithms” and “GDP Prediction and Forecasting using Machine Learning,” which allowed us to compare our model’s performance against theirs as shown in Table 5. In our study, “GDP Prediction of The Gambia using Generative Adversarial Networks” utilizing the Generative Adversarial Networks, we achieved a Mean Absolute Error (MAE) of 0.0499, Root Mean Squared Error (RMSE) of 0.0617, and an R^2^ score of 0.9968. Compared to the study “GDP Growth Prediction of Bangladesh using Machine Learning Algorithms” (Random Forest Regressor), which reported an MAE of 0.06267, RMSE of 0.068084, and R^2^ of 0.750, our model demonstrates significantly higher predictive accuracy, as reflected by the higher R^2^ and relatively low error metrics.

Similarly, when compared with “GDP Prediction and Forecasting using Machine Learning” (Gradient Boosting), which reported an MAE of 2362.935, RMSE of 3469.360, and an R^2^ of 0.889, our model outperforms theirs in terms of all evaluation metrics. Our lower MAE and RMSE values indicate a much better fit of our model, while the R^2^ score of 0.9971 highlights its strong predictive capability. These comparisons underscore the efficiency of our K-Nearest Neighbor Regressor model in accurately predicting GDP growth for The Gambia, with superior performance metrics relative to the referenced studies. Additionally, while the study by Evholt and Larsson (2020) used GANs for unemployment prediction, their model demonstrated a significant improvement over ARIMA, with RMSE results showing promising potential in forecasting more volatile metrics like the S&P 500. This comparison underscores the efficiency of our GAN-based approach in accurately predicting GDP growth for The Gambia, outperforming all referenced studies.

**Table 5:** Comparison of model performance across different studies.

Author	Work	Algorithm name	MAE	RMSE	R^2^
Our work	GDP Prediction of The Gambia using Generative Adversarial Networks	Generative adversarial networks	0.0499	0.0543	0.9968
Hossain et al. (2021)	Gdp Growth Prediction of Bangladesh using Machine Learning Algorithms	Random forest regressor	0.06267	0.068084	0.750
Gharte et al. (2022)	GDP Prediction and Forecasting using Machine Learning	Gradient boosting	2362.935	3469.360	0.889

## 6 Discussions

The evaluation of machine learning models—Generative Adversarial Networks (GANs), Random Forest (RF), Support Vector Machine (SVM), and XGBoost—highlighted their unique strengths in predicting GDP growth for The Gambia over the period 1970–2022. The analysis emphasized forecasting performance, interpretability of results, and their applicability in modeling economic variables.

### 6.1 Predictive accuracy

The primary assessment criterion was each model’s ability to predict GDP growth, evaluated using performance metrics such as Mean Absolute Error (MAE), Root Mean Squared Error (RMSE), Mean Absolute Percentage Error (MAPE), and R-squared (R^2^). Among the models, Generative Adversarial Networks (GANs) emerged as the most accurate, achieving an exceptional R^2^ score of 0.9968, indicating it explained nearly all the variance in GDP growth. This was followed closely by Random Forest (R^2^) and XGBoost (R^2^ = 0.9843). Conversely, the Support Vector Machine (SVM) performed poorly, with a negative R^2^ value of −0.0246 in most configurations, underscoring its limited suitability for GDP forecasting.

The GAN model demonstrated unparalleled predictive accuracy by effectively capturing complex, nonlinear relationships within the data and leveraging its synthetic data generation capabilities to enhance generalization. While XGBoost and Random Forest also performed well, their R^2^ scores, though high, showed slightly larger deviations during periods of rapid economic change. Additionally, while the.

differences in MAE and RMSE between Random Forest and XGBoost were minor, the GAN model consistently outperformed both in terms of predictive precision. Overall, the analysis underscored the GAN model’s superiority in forecasting GDP growth trends, particularly in addressing data complexity and variability. However, Random Forest and XGBoost remained strong alternatives, balancing accuracy with greater interpretability and practical utility.

### 6.2 Interpretability of results

While predictive accuracy is a cornerstone of economic modelling, the interpretability of results is equally critical. In this analysis, the features identified through Pearson Correlation analysis offered valuable insights into the relative importance of various economic factors influencing GDP growth. Among these, government expenditure emerged as the most significant driver, with a correlation coefficient of 0.921720, highlighting its pivotal role in shaping GDP per capita. Other key contributors included agroforestry and fishing value (0.882439) and imports (0.824819), reflecting the substantial impact of economic sectors and trade dynamics on growth. Variables such as urban population (0.812646) and exports (0.814244) also demonstrated strong correlations, providing additional insights into demographic and trade-related influences on economic performance. The interpretability offered by this feature selection approach is particularly beneficial for policymakers, enabling them to identify and prioritize critical areas for intervention. For instance, understanding the dominant influence of government expenditure can guide resource allocation strategies, while insights into trade and population dynamics can inform policies to bolster economic resilience and growth. This ability to clearly delineate feature importance makes Pearson Correlation analysis an invaluable tool for economic forecasting and decision-making, bridging the gap between statistical findings and actionable insights.

### 6.3 Applicability in economic prediction

This evaluation also examined the practicality and relevance of machine learning models for real-world economic forecasting. The scenario analysis highlighted the Random Forest (RF) model’s effectiveness in adapting to dynamic economic conditions. The model demonstrated robust performance in predicting GDP growth under various scenarios, such as shifts in export–import dynamics and urbanization trends. This adaptability is particularly valuable in economic forecasting, where unpredictable changes require models capable of accommodating diverse conditions. For example, in Scenario 1 (High Exports, Low Imports), the RF model projected a moderate increase in GDP growth, showcasing the potential benefits of favorable trade balances. In contrast, Scenario 2 (High Urban Population, Low Government Expenditure) revealed the mixed effects of urbanization combined with reduced public spending, highlighting potential constraints on economic growth. These findings offer critical insights for policymakers, providing data-driven guidance for resource allocation and strategic planning.

Moreover, the scenario analysis underscored the RF model’s utility in informing policy decisions. For instance, predictions of reduced GDP growth under conditions of low government expenditure emphasize the need for sustained public investment. Similarly, the model’s forecast of economic gains from increased exports and reduced imports supports policies aimed at enhancing trade competitiveness. By enabling the simulation and analysis of hypothetical scenarios, the RF model proves to be a valuable tool for developing policies that strengthen economic resilience and foster sustainable growth.

### 6.4 Model limitations

Generative Adversarial Networks (GANs) showcased remarkable predictive accuracy by effectively addressing data sparsity and capturing nonlinear relationships in economic forecasting. The incorporation of synthetic data generation significantly improved model performance, setting a promising benchmark for future studies in similar domains. However, the reliance on annual data in this research limits the ability to capture short-term economic fluctuations, reducing the granularity of forecasts. Additionally, the high computational complexity and prolonged training times of GANs pose challenges for real-time applications, making them less practical in scenarios requiring rapid predictions. Their intricate architecture also diminishes interpretability compared to Pearson Correlation analysis or models like Random Forest (RF), which may hinder their acceptance in policymaking, where transparency and feature importance analysis are crucial.

While the Random Forest model demonstrated excellent predictive accuracy and interpretability, its computational demands present a notable drawback for real-time forecasting. As the size of the dataset and the number of features increase, the model’s training time and resource requirements escalate significantly, reducing its suitability for scenarios requiring quick predictions. Moreover, like all data-driven models, RF relies heavily on historical data, which limits its ability to predict unprecedented economic events, such as those caused by major policy changes or global crises. These limitations highlight the need to complement machine learning models with expert judgment and alternative forecasting approaches in such situations.

The Support Vector Machine (SVM) model, despite its theoretical robustness, faced substantial challenges when dealing with sparse and noisy data, as evidenced by its poor performance metrics. While SVM may perform well with highly structured datasets, its application in economic forecasting—especially in contexts involving small, irregular datasets like those of The Gambia—appears to be limited.

## 7 Conclusions and future work

The GDP, which provides information about the size and durability of a country’s economy, is a crucial indicator for evaluating its economic health. The real GDP growth rate is a measure of the economy’s overall performance. While a fall in GDP identifies areas of concern, an increase in GDP indicates economic development. Forecasting GDP changes is essential for determining which industries need to be intervened in to promote economic expansion.

A number of factors, such as high debt levels, low revenue generation, and significant reliance on foreign aid affect economic stability in The Gambia. Recent projections indicate that without substantial reforms, the country will face increasing fiscal deficit. The aim of this study was to create a machine learning-based model that would predict GDP growth using World Bank Development Indicators historical data from 1970 to 2022. With GDP as the dependent variable, the dataset has 14 independent variables, including remittances, foreign direct investment, official development assistance, exports, imports, inflation, and government spending. The study highlights the potential of machine learning models, particularly Generative Adversarial Networks (GANs) and Random Forests, to provide highly accurate and interpretable forecasts of GDP growth. These models are valuable tools for economic prediction due to their ability to handle complex, non-linear relationships and offer actionable policy insights.

To identify the top-performing model for GDP prediction, we investigated a number of machine learning methods, such as Generative Adversarial Networks, Random Forest, XGBoost, and Support Vector Machine (SVM). Metrics including RMSE, MAE, MPAE and R2 were used to assess the model’s performance. All the algorithms showed higher accuracy, but Generative Adversarial Networks had the highest accuracy, which achieved 99% prediction accuracies. The most dependable model for capturing intricate correlations between GDP and its affecting components, however, RF and XGBoost, also achieved an accuracy of 98% each. These overall highlights the potential of integrating ML models to improve real-time GDP growth forecasting.

Future research could explore applying GANs to other economic indicators, such as inflation or unemployment rates, to assess their broader applicability in economic forecasting. Incorporating higher- frequency data (e.g., quarterly or monthly) and experimenting with hybrid architectures that combine GANs with other deep learning techniques may further enhance predictive accuracy and scalability. Future research could also explore incorporating real-time data and hybrid models to further refine and improve these forecasts. Further work would consider the use of real-time information and mixed models to improve these estimates. We also intend to improve the model in subsequent research by including more factors that might have a big influence on GDP, like the dynamics of tourism and diaspora investment. Also, we want to explore the prediction accuracy for GAN by using neural networks like LSTM and CNN for the generator and discriminator in our subsequent research. Furthermore, improve the prediction framework so that economists can more easily utilize machine learning techniques, enabling data-driven policymaking. Our method, which focuses on causal pathways, will enhance GDP forecasting and aid in the creation of policies that support The Gambia’s sustainable economic growth.

## Data Availability

Publicly available datasets were analyzed in this study. This data can be found here: https://databank.worldbank.org/source/2?country=IRN&l=en.
