# Supplementary material for: GDP prediction of The Gambia using generative adversarial networks
Source: Front Artif Intell. 2025 Mar 5;8:1546398. doi: 10.3389/frai.2025.1546398 (PMC11920123; doi:10.3389/frai.2025.1546398)
Supplement: Supplementary file 1 [file Table_1.docx]

S1: Dataset Description.

| **Identifier** | **Attribute** | **Frequency** | **Measure** |
| --- | --- | --- | --- |
| y | Gross Domestic Product ($ per capital) | Y | current US$ |
| x1 | Personal remittances | Y | current US$ |
| x2 | Foreign direct investment | Y | current US$ |
| x3 | Government Expenditure | Y | current US$ |
| x4 | Official aid received | Y | current US$ |
| x5 | Inflation | Y | Percent |
| x6 | Export of Goods and Services | Y | current US$ |
| x7 | Import of Goods and Services | Y | current US$ |
| x8 | Net Migration | Y | Percent |
| x9 | Agroforestry and fishing value | Y | current US$ |
| x10 | Secondary education, general pupils | Y | Percent |
| x11 | Unemployment | Y | Percent |
| x12 | Urban population | Y | Percent |
| x13 | Total Population | Y | Percent |

S2: Recent Methods for GDP Forecasting in Various Nations.

| **Author** | **Method** | **Nation** |  | **Duration** | **Outcomes** |
| --- | --- | --- | --- | --- | --- |
| Our Research | GDP Prediction using  Generative Adversarial | Gambia |  | 1970 to 2022 | Achieved the highest  R^2^ score of 99% |
|  | Networks |  |  |  |  |
| Shams et al. (2024) | PC-LSTM-RNN  (Pearson Correlation | India |  | 1961 to 2021 | Achieved the highest  R^2^ value of 99.99% |
|  | LSTM RNN) |  |  |  |  |
| Vuleti´c et al. (2024) | Fin-GAN (Generative | Global | (Equity | Historical data | Fin-GAN achieved |
|  | Adversarial Networks | data) |  |  | higher Sharpe Ratios |
|  | for financial forecast- |  |  |  | than LSTMs and |
|  | ing) |  |  |  | ARIMA |

Kant et al. (2024) Random Forest Netherlands 1992 to 2018 Random Forest pro-

vided the most accu- rate forecast and now- casts

Naaz et al. (2024) Random Forest, Neu-

ral Network regression

Global Recent years Both models showed strong predictive capa- bilities for GDP per capita

Koch et al. (2024) Elastic Net Regression Historical data

(Europe and North America)

1300 to 1800 Achieved 90% variance

explanation for histori- cal GDP per capita es- timates

Thilaka and Sundar- avalli (2024)

Decision Tree, Sup- port Vector Regres- sion, Polynomial Re- gression

Global (Gap- minder dataset)

Recent years Decision Tree Regres-

sion achieved the best R-squared value of 1

Ahammad et al. (2024) KNN, Random For-

est, AdaBoost, Deci- sion Tree

Zhang et al. (2023) WA-LSTM (Wavelet

Analysis LSTM)

Bangladesh 1976 to 2020 KNN achieved the

highest accuracy of 98.40%

China Quarterly data Outperformed bench- mark models for quar- terly GDP forecasting

Srinivasan et al. (2023) Polynomial Regression India Recent years Polynomial regression

achieved 91% accuracy in predicting GDP

Lai (2022) Particle Swarm Opti- mization (PSO) and Elman NN

China 1992 to 2020 MAPE: 0.0236,

RMSE: 0.0166

Velidi (2022) LSTM and RNN Indonesia 2018 to 2022 Achieved accuracy of

80%-90%

Longo et al. (2022) RNN, DFM-GAS United States Post-2008-09

crisis

RNN and DFM-GAS

improve forecasts

Hossain et al. (2021) Random Forest Re-

gressor

Muchisha et al. (2021) Random Forest, Ridge,

LASSO, Elastic Net, SVM

Bangladesh 1980 to 2019 MSE: 0.004, MAE:

0.062, RMSE: 0.068

Indonesia 2013 to 2019 Random Forest out-

performed other mod- els

Maccarrone et al. (2021)

K-Nearest Neighbour (KNN)

U.S.A. 1976 to 2020 KNN outperformed traditional time series methods for long-term forecasting

**References**

Md Saymon Ahammad, Sadia Akter Sinthia, Mahjabeen Hossain, Md Mustak Ahmed, Md Nurul Afsar Ikram, et al. Machine learning for gdp forecasting: Enhancing economic projections in bangladesh. In *2024 15th International Conference on Computing Communication and Networking Technologies (ICCCNT)*, pages 1–5. IEEE, 2024.

Amman Hossain, Md Hossen, Md Mahmudul Hasan, and Abdus Sattar. Gdp growth prediction of bangladesh using machine learning algorithm. In *2021 Third International Conference on Intelligent Communication Technologies and Virtual Mobile Networks (ICICV)*, pages 812–817. IEEE, 2021.

Dennis Kant, Andreas Pick, and Jasper de Winter. Nowcasting gdp using machine learning methods. *AStA Advances in Statistical Analysis*, pages 1–24, 2024.

Philipp Koch, Viktor Stojkoski, and C´esar A. Hidalgo. Augmenting the availability of historical gdp per capita estimates through machine learning. *Proceedings of the National Academy of Sciences*, 121(39):e2402060121, 2024.

Han Lai. A comparative study of different neural networks in predicting gross domestic product. *Journal of Intelligent Systems*, 31(1):601–610, 2022.

Luigi Longo, Massimo Riccaboni, and Armando Rungi. A neural network ensemble approach for gdp forecasting.

*Journal of Economic Dynamics and Control*, 134:104278, 2022.

Giovanni Maccarrone, Giacomo Morelli, and Sara Spadaccini. Gdp forecasting: Machine learning, linear or autore- gression? *Frontiers in Artificial Intelligence*, 4:757864, 2021.

Nadya Dwi Muchisha, Novian Tamara, Andriansyah Andriansyah, and Agus M Soleh. Nowcasting indonesia’s gdp growth using machine learning algorithms. *Indonesian Journal of Statistics and Its Applications*, 5(2):355–368, 2021.

Saifeen Naaz, Himanshu Pandey, and C Lakshmi. Forecasting gdp per capita using machine learning algo- rithms. In *2024 Second International Conference on Emerging Trends in Information Technology and Engineering (ICETITE)*, pages 1–5. IEEE, 2024.

Mahmoud Y Shams, Zahraa Tarek, El-Sayed M El-kenawy, Marwa M Eid, and Ahmed M Elshewey. Predicting gross domestic product (gdp) using a pc-lstm-rnn model in urban profiling areas. *Computational Urban Science*, 4(1):3, 2024.

Nikhil Srinivasan, M Krishna, V Naveen, SM Kishore, Sampath Kumar, and R Subha. Predicting indian gdp with machine learning: a comparison of regression models. In *2023 9th International Conference on Advanced Computing and Communication Systems (ICACCS)*, volume 1, pages 1855–1858. IEEE, 2023.

A Thilaka and E Sundaravalli. A machine learning approach to gdp prediction by analyzing economic indica- tors. In *2024 2nd International Conference on Artificial Intelligence and Machine Learning Applications Theme: Healthcare and Internet of Things (AIMLA)*, pages 1–7. IEEE, 2024.

Gurunadh Velidi. Gdp prediction for countries using machine learning models. *Journal of Emerging Strategies in New Economics*, 1(1):41–49, 2022.

Milena Vuleti´c, Felix Prenzel, and Mihai Cucuringu. Fin-gan: Forecasting and classifying financial time series via generative adversarial networks. *Quantitative Finance*, 24(2):175–199, 2024.

Yaling Zhang, Wenying Shang, Na Zhang, Xiao Pan, and Bonan Huang. Quarterly gdp forecast based on coupled economic and energy feature wa-lstm model. *Frontiers in Energy Research*, 11:1329376, 2023.
